# The JAK2/STAT3 inhibitor pacritinib effectively inhibits patient-derived GBM brain tumor initiating cells *in vitro* and when used in combination with temozolomide increases survival in an orthotopic xenograft model

**DOI:** 10.1371/journal.pone.0189670

**Published:** 2017-12-18

**Authors:** Katharine Victoria Jensen, Orsolya Cseh, Ahmed Aman, Samuel Weiss, Hema Artee Luchman

**Affiliations:** 1 Hotchkiss Brain Institute and Department of Cell Biology and Anatomy, University of Calgary, Calgary, Alberta, Canada; 2 Drug Discovery Program, Ontario Institute for Cancer Research, Toronto, Ontario, Canada; Northwestren University, UNITED STATES

## Abstract

**Purpose:**

The prognosis for patients diagnosed with glioblastoma multiforme (GBM) remains dismal, with current treatment prolonging survival only modestly. As such, there remains a strong need for novel therapeutic strategies. The janus kinase (JAK)2/signal transducer and activator of transcription (STAT)3 pathway regulates many cellular processes in GBM, including survival, proliferation, invasion, anti-apoptosis, and immune evasion. Here, we evaluated the preclinical efficacy of pacritinib, a novel compound targeting JAK2, using a collection of diverse patient-derived brain tumor initiating cells (BTICs).

**Experimental design:**

The effects of pacritinib on BTIC viability and sphere forming capacity were evaluated *in vitro* using the alamarBlue and neurosphere assays, respectively. On-target inhibition of JAK2/STAT3 signaling was investigated using western blotting. The efficacy of pacritinib was tested *in vivo* in pharmacokinetic analyses, liver microsome analyses, and Kaplan-Meier survival studies.

**Results:**

*In vitro*, pacritinib decreased BTIC viability and sphere forming potential at low micromolar doses and demonstrated on-target inhibition of STAT3 signaling. Additionally, pacritinib was found to improve the response to temozolomide (TMZ) in TMZ-resistant BTICs. *In vivo*, systemic treatment with pacritinib demonstrated blood-brain barrier penetration and led to improved overall median survival in combination with TMZ, in mice orthotopically xenografted with an aggressive recurrent GBM BTIC culture.

**Conclusion:**

This preclinical study demonstrates the efficacy of pacritinib and supports the feasibility of testing pacritinib for the treatment of GBM, in combination with the standard of care TMZ.

## Introduction

Glioblastoma multiforme (GBM), a World Health Organization (WHO) grade IV astrocytoma, is the most common and lethal central nervous system tumor [1,2]. It is a rare disease, with 2–4 new GBM diagnoses per 100,000 in North America every year. The incidence of the disease peaks among those aged 45 to 75 [2]. It is estimated that 12,390 new cases of GBM will be diagnosed in the United States in 2017 [3]. The current standard of care involves maximal safe surgical resection of the tumor followed by treatment with temozolomide (TMZ) chemotherapy and/or radiotherapy [4]. Treatment only modestly improves the outcome of GBM patients, as median survival remains only 14.6 months following diagnosis [1,4]. GBM is lethal as it inevitably recurs despite aggressive treatment strategies. Disease recurrence has been postulated to be due to the presence of brain tumor initiating cells (BTICs). BTICs have the cancer stem cell properties of long-term self-renewal, multi-lineage differentiation, and the capability to readily initiate tumors in mice that are similar to the human GBMs from which they were derived [5,6]. Therefore, in order to prevent post-treatment recurrence, targeting BTICs is likely a crucial therapeutic strategy to make this devastating disease more manageable.

Our improved understanding of GBM biology has identified key signaling pathways that may be exploited for molecular-based therapeutics [7]. An increasing body of evidence points to the signal transducer and activator of transcription 3 (STAT3) [8–11] as one such oncogenic signaling hub. In the central nervous system, the janus kinase (JAK)2/STAT3 pathway is highly active during embryonic development [12]. During adulthood, the activity of JAK2/STAT3 pathway is dramatically reduced. However, JAK2/STAT3 signaling becomes deregulated in GBM and is important for tumorigenesis [9,13,14]. One mechanism through which the JAK2/STAT3 pathway is activated in GBM is through upstream receptor tyrosine kinases (RTKs) such as the epidermal growth factor receptor (EGFR), which itself is highly mutated in GBMs [7,15]. Activated STAT3 is present at high levels in GBMs and patient-derived BTICs [9,16,17]. GBM patients with high levels of activated STAT3 have a more aggressive disease and poorer clinical outcomes compared to those with low levels of activated STAT3 [9]. Due to the role of STAT3 in tumorigenesis and the abnormal activation of the pathway in several cancer types (reviewed in [11], [18], and [19]) including GBM, the JAK2/STAT3 pathway has attracted considerable attention as a potential novel therapeutic target.

Several small-molecule inhibitors have been developed to target JAK2, an upstream regulator of STAT3. JAK2 phosphorylates cell surface receptors on tyrosine residues creating docking sites for STAT3 proteins. Once STAT3 is recruited to the receptor, it is phosphorylated by JAK2 allowing STAT3 to form a dimer and move into the nucleus where it can activate the transcription of target genes (reviewed in [19]). We have previously demonstrated that inhibiting the JAK2/STAT3 pathway in GBM BTICs using the JAK2 inhibitors, WP1066 and Cucurbitacin-I, led to on-target inhibition of downstream STAT3 signaling, decreased the viability of molecularly heterogeneous BTICs *in vitro*, and improved survival in an orthotopic BTIC xenograft mouse model [17]. This previous study demonstrated that targeting the JAK2/STAT3 pathway was an effective means to decrease BTIC survival, proliferation, and tumorigenicity. A phase I clinical trial with WP1066 is currently underway for brain metastasis from melanoma and recurrent glioma (NCT01904123) [20]. However, there remains a need to identify new compounds that have good pharmacokinetic properties and promising safety profiles that may be amenable treatment strategies for GBM.

We tested the efficacy of pacritinib, a JAK2 inhibitor currently in phase 3 trials for myelofibrosis [21], on our large collection of molecularly diverse patient-derived BTIC cultures. We report that, *in vitro*, pacritinib dramatically decreased viability and sphere forming capacity in a panel of BTICs. Pacritinib was also found to improve the efficacy of TMZ, specifically in TMZ-unresponsive BTIC cultures with unmethylated *MGMT* (O-6-methylguanine-DNA methyltransferase) promoters. *In vivo*, systemic treatment with pacritinib was tolerated and demonstrated favourable pharmacokinetic properties. While pacritinib was found to be unstable in mouse liver microsomes, the drug was stable in human liver microsomes. Despite the rapid metabolism of pacritinib, there was a significant increase in overall median survival in combination with TMZ in mice orthotopically xenografted with an aggressive recurrent GBM BTIC culture. These results suggest that benefits observed in our mouse model may hold further promise in humans, where the drug is not as rapidly metabolized and has promising safety profiles.

## Materials and methods

### Brain tumor initiating cell culture

Following informed consent from GBM patients, GBM BTICs were cultured from tumor specimens obtained during operative procedures as previously described [17,22–24] and approved by the University of Calgary Ethics Review Board and the Health Research Ethics board of Alberta—Cancer Commitee (HREBA). Briefly, serum-free medium (SFM) was used to initiate BTIC cultures. Non-adherent spheres formed after 7–21 days in culture and were expanded, then cryopreserved in 10% dimethyl sulfoxide (DMSO; Sigma-Aldrich) in SFM until used in experiments.

### BTIC viability and neurosphere assays

Accumax (eBioscience) was used to enzymatically dissociate BTIC spheres to single cells. Cells were seeded at 1000 cells per well in 96-well plates and treated with vehicle (DMSO) or drug, 24 hours after plating. BTIC viability was assessed after 7–14 days, once spheres were formed in control wells, by performing the alamarBlue^™^ (Invitrogen) assay per the manufacturer’s instructions. In brief, the alamar dye was added to each well and plates were read 6 hours later. Fluorescence intensity (excitation 540 nm; emission 590 nm) was measured on a SpectraMax M Series Multi-Mode Microplate reader. The neurosphere assay was used to assess drug sensitivity. Cells were plated at a density of 500–1500 cells per well in 96-well plates, treated with vehicle (DMSO) or drug. The number and size of the spheres were quantified 7–21 days later. All experiments were performed in triplicate with a minimum of 3 wells per condition.

### Bliss independence analyses

BTICs were treated with suboptimal doses of 1 μM pacritinib (CTI Biopharma), 10 μg/mL TMZ (Sigma), or a combination of pacritinib and TMZ. Once spheres had formed in the control conditions, alamarBlue was added and incubated for 6 hours and fluorescence intensity (excitation 540 nm; emission 590 nm) was measured on a SpectraMax M Series Multi-Mode Microplate reader. Percent inhibition was determined by normalizing each well to the control well (0.1% DMSO). In order to determine if there was synergy between pacritinib and TMZ, the bliss independence analysis was used. The following equation was used to determine the bliss expectation: E = (A+B)—(A × B), where A and B are the fractional growth inhibitions of drug A (pacritinib) and B (TMZ) at a given dose. A combination is synergistic when a combination effect above the bliss expectation value is observed [25,26].

### Western blotting

BTIC spheres were enzymatically dissociated as described above and plated at 10^6^ cells/2 mL of media. Cells were treated with vehicle (DMSO), pacritinib, TMZ, or a combination of pacritinib and TMZ and pelleted at select time points (3, 24, and 48 hours). For protein extraction, BTICs were lysed in modified radio-immunoprecipitation assay buffer supplemented with Complete Mini protease (Roche) and Halt phosphatase (Thermo Scientific) inhibitor cocktails. Protein concentrations were quantified using the BioRad protein assay; 20 μg of protein were loaded on 6% or 10% sodium dodecyl sulfate polyacrylamide gel electrophoresis (SDS PAGE) gels and transblotted to nitrocellulose membranes. Blots were stained with primary antibodies followed by horseradish peroxidase-conjugated secondary antibodies. Primary antibodies included p-STAT3 Y705 (1:1000; Cell Signaling Technology), STAT3 (1:1000; Cell Signaling Technology), p-Akt S473 (1:1000; Cell Signaling Technology), Akt (1:1000; Cell Signaling Technology), p-p44/42 MAPK (T202/Y204) (1:1000; Cell Signaling Technology), p44/42 MAPK (1:4000; Cell Signaling Technology), poly (ADP-ribose) polymerase (1:1000; Cell Signaling Technology), β-tubulin (1:1000; Cell Signaling Technology), and Actin (1:1000; Santa Cruz Biotechnology). Secondary antibodies included donkey anti-mouse (1:5000; Cell Signaling Technology), donkey anti-goat (1:5000; Millipore), and goat anti-rabbit (1:5000; Cell Signaling Technology). The blots were washed with tris-buffered saline and tween 20 (T-TBS) (50mM Tris, 150mM NaCl, 0.05% Tween 20, pH 7.6) and blocked with tris-buffered saline (TBS) before imaging. Bands were visualized with the SuperSignal West Pico chemiluminescent solution (Thermo Scientific) and an Amersham^™^ Imager 600 (General Electric).

### Pharmacokinetic analyses of pacritinib by liquid chromatography-mass spectrometry

Animal studies were performed following institutional ethical guidelines and protocols approved by the University of Calgary Health Sciences Animal Care Committee, accredited by the Canadian Council on Animal Care (CCAC). Non-tumor bearing mice were treated with 100 or 200 mg/kg doses of pacritinib by oral gavage for five consecutive days. Three mice per group were used. Post-dosing, blood was collected at 30 and 300 minute time points. The serum was separated by centrifugation. Liquid chromatography-mass spectrometry (LC-MS) was performed to determine the serum concentration of pacritinib. To determine brain penetration, brains were harvested and processed at the 300 minute time point following transcardiac perfusion with PBS. Brains were removed and flash frozen at -80°C. Pacritinib accumulation in the brain was analyzed using LC-MS.

### Mouse and human microsomal stability assay

For Phase I analysis, test compounds (10 mM stock in DMSO) were incubated at a final concentration of 1 μM (this concentration assumed to be well below the K_m_ values to ensure linear reaction conditions). Working stocks were initially diluted to a concentration of 40 μM in 0.1 M potassium phosphate buffer before addition to the reaction vials. Pooled mouse (CD-1, male) or human (50 donors) liver microsomes were utilized at a final concentration of 0.5 mg/mL. Duplicate wells were used for each time point (0 and 30 minutes). Reactions were carried out at 37°C in a shaker, and the final concentration of DMSO was kept constant at 0.01%. The final volume for each reaction was 100 μL, which included the addition of an NADPH-Regeneration solution (NRS) mix. The NRS mix was comprised of glucose 6-phosphate dehydrogenase (0.4 U/mL), NADP+ (1.3 mM), MgCl_2_ (3.3 mM), and glucose 6-phosphate (3.3 mM) in assay mixtures. Upon completion of the 30-minute time point, reactions were terminated by the addition of 1.5-volumes (150 μL) of ice-cold acetonitrile with 0.5% formic acid and internal standard. Samples were then centrifuged at 4,000 rpm for 10 minutes to remove debris and precipitated protein. Approximately 150 μL of supernatant was subsequently transferred to a new 96-well microplate for LC-MS analysis. Narrow-window mass extraction LC-MS analysis was performed for all samples using a Waters Xevo quadrupole time-of-flight (QToF) mass spectrometer and an ACQUITY UPLC system, to determine relative peak areas of parent compound.

### Intracranial BTIC xenografts

For Kaplan-Meier survival studies, BTIC spheres from BT53 or BT147 were dissociated to a single-cell suspension. Mice were anaesthetized using 10 mg/kg ketamine/xylazine. Buprenorphine (0.05 mg/kg) was administered once pre-operatively and once post-operatively to minimize animal suffering. 5 x 10^4^ cells were stereotactically implanted into the right striata of 8- to 10-week-old female severe combined immunodeficient (SCID) mice, as previously described [17,22–24] and approved by the University of Calgary Health Sciences Animal Care Committee, accredited by CCAC. Mice were randomized to vehicle or treatment cohorts, with a minimum of 8 mice per group, one week after cell implantation. For BT147, vehicle (Ora-Plus; Galenova), pacritinib (100 mg/kg), TMZ (30 mg/kg), or a combination of pacritinib (100 mg/kg) and TMZ (30 mg/kg), were administered via oral gavage. Animals completed a five-week regimen of three weekly treatments for a total of 15 treatments. For BT53, 100 mg/kg of pacritinib was administered twice per day alone or in combination with 50 mg/kg TMZ once per day, for one week via oral gavage. Following this, mice received 100 mg/kg pacritinib twice per day in combination with 10 mg/kg TMZ once per day for two weeks. Mice were euthanized with a lethal dose of sodium pentobarbital at a pre-defined humane endpoint. Specific criteria for the endpoint included: 20% weight loss, lack of grooming, inactivity, hind limb paralysis, and/or inability to eat or drink. The body weights were closely monitored throughout the course of the experiment. Mice were monitored daily and there were no unexpected deaths reported in this study. The presence of tumor was confirmed in the mice via necropsy and cranial dissection.

### Microscopy and statistical analyses

Images of BTIC cultures were captured using a Zeiss Axiovert 40 CFL inverted microscope and AxioVision software or the IncuCyte Zoom (Essen Bioscience). An Olympus Slide Scanner was used to image the brain sections at the University of Calgary Hotchkiss Brain Institute Core Facility. OlyVia (Olympus Life Science) software was used to analyze the images. Statistically significant differences between control and treated BTIC groups were evaluated by means of analysis of variance (ANOVA). Data are illustrated in bar graphs, including mean ± SEM. Asterisks denote statistical significance. For Kaplan-Meier survival studies, statistical difference in median survival was determined by the log-rank test. For *in vivo* microsomal stability analyses, data are illustrated in bar graphs, including mean ± SD. All analyses were performed using GraphPad Prism Version 6.0.

## Results

### On-target inhibition of JAK2/STAT3 signaling with pacritinib effectively decreases viability and sphere formation potential of molecularly diverse BTICs *in vitro*

**Fig 1:**
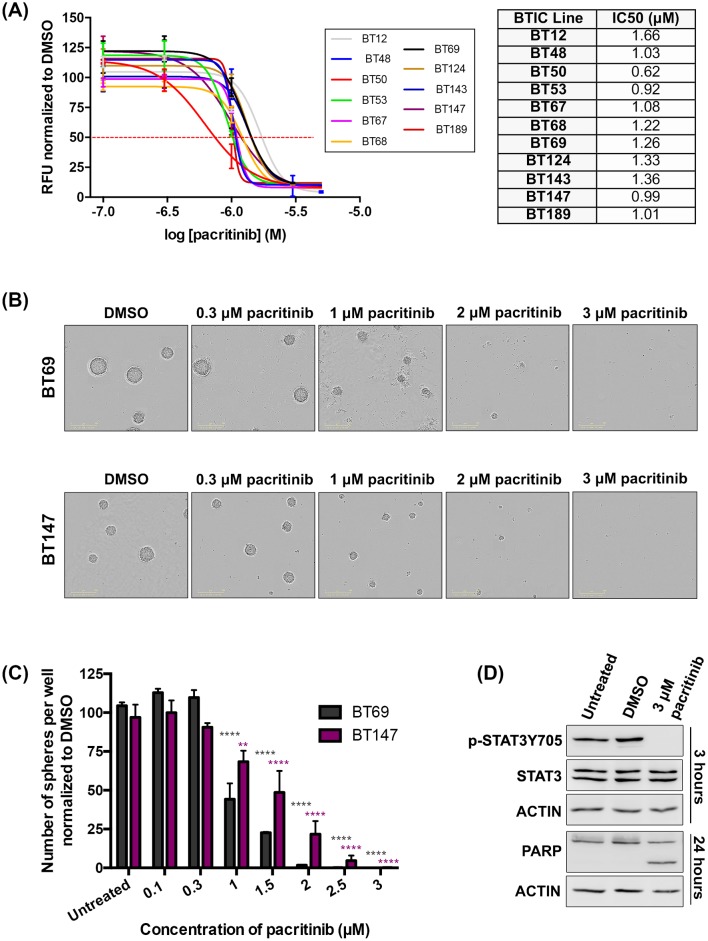
Pacritinib effectively decreases BTIC viability and sphere forming capacity and has on-target activity on phospho-STAT3. (A) Pacritinib dramatically decreased cell viability in a dose dependent manner in eleven molecularly diverse patient-derived BTIC cultures with IC_50_ values from 0.62 μM to 1.66 μM. Pacritinib decreased sphere formation in a dose dependent manner. Representative images (B) and quantifications (C) are shown for two representative BTIC cultures (BT69 and BT147). Pacritinib completely abolished sphere formation at 3 μM in BT69 and BT147 (**** denotes p < 0.0001 vs untreated; ANOVA). Error bars represent SEM. (D) Pacritinib had on-target activity as seen by a decrease in the phosphorylation of tyrosine 705 at 3 hours. It also resulted in increased cell death as seen by an increase in the cleaved PARP at 24 hours (representative line BT69 shown).

We first asked whether pacritinib was an effective inhibitor of GBM BTIC viability. We assembled a panel of eleven patient-derived molecularly heterogeneous BTIC cultures with previously reported status of common GBM molecular alterations including *MGMT* promoter methylation, *EGFR*, *PTEN*, *TP53*, *NF1*, *IDH1*, and *CDKN2A* mutations [24,27] (S1 Table). Pacritinib reduced cell viability in all BTIC cultures tested in a dose dependent manner (Fig 1A), as measured by the alamarBlue assay. The IC_50_ values for the eleven BTIC cultures tested ranged from 0.62 μM to 1.66 μM. Viability was reduced by 50% at 1.5 μM of pacritinib in 10/11 BTIC cultures tested (Fig 1A). Similar results were obtained using a neurosphere assay, whereby pacritinib induced a dose-dependent decrease in the number of neurospheres formed (representative BTIC cultures, BT69 and BT147, shown; Fig 1B and 1C). Both sphere size and number decreased with increasing drug concentration (Fig 1B and 1C, S1 Fig). A concentration of 5 μM pacritinib was sufficient to completely inhibit sphere formation in all BTIC cultures tested (S1 Fig). Moreover, pacritinib decreased activated STAT3 at 3 hours as seen by reduced phosphorylation on tyrosine 705 (representative line BT69 shown; Fig 1D). Pacritinib also increased cell death as seen by an increase in cleaved PARP levels at the 24-hour time point (Fig 1D). Pacritinib-mediated JAK2/STAT3 inhibition thus appears to be highly effective at decreasing BTIC viability and sphere-formation *in vitro* in all BTIC cultures tested, independent of their molecular profiles.

### Pacritinib does not attenuate BTIC sensitivity to TMZ

**Fig 2:**
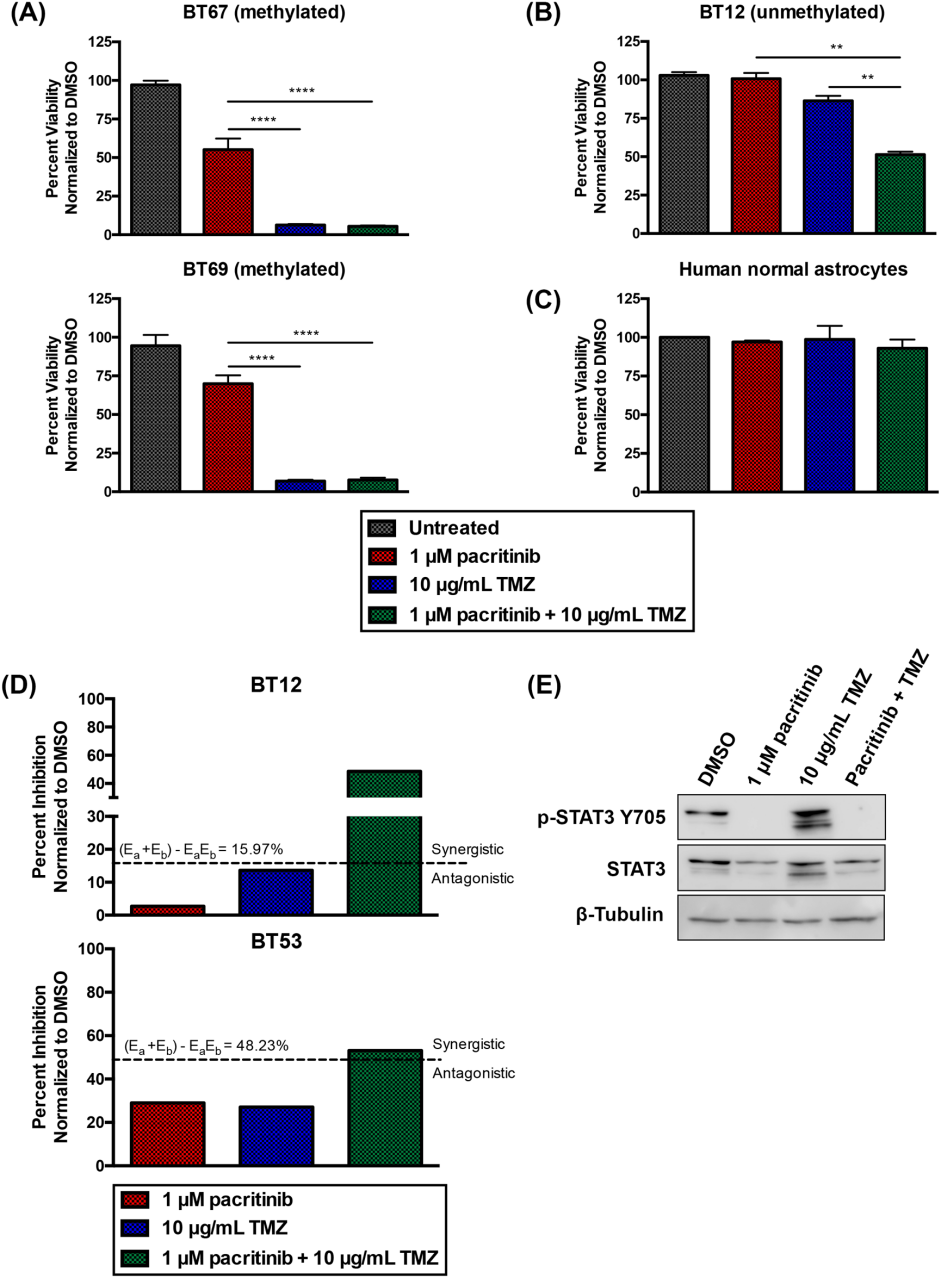
Pacritinib does not attenuate BTIC sensitivity to TMZ. (A) The *MGMT* methylated BTIC cultures were highly sensitive to TMZ. Pacritinib did not reverse the effectiveness of TMZ in these methylated BTICs (representative BTIC cultures, BT67 and BT69, shown; **** denotes p < 0.0001; ANOVA). Error bars represent SEM. (B) *MGMT* unmethylated BTIC cultures were largely resistant to TMZ, but responded to the combination of pacritinib and TMZ (representative BTIC culture BT12 shown; ** denotes p < 0.003; ANOVA). Error bars represent SEM. (C) The combination of pacritinib and TMZ had no effect on normal human astrocytes. (D) Bliss independence analysis shows that suboptimal doses of pacritinib and TMZ are synergistic in BTICs (representative BTIC cultures, BT12 and BT53, shown). (E) 1 μM pacritinib dramatically reduced phosphorylation of tyrosine 705 of STAT3 (p-STAT3 Y705) in BT53 at 48 hours. Treatment with 10 μg/mL TMZ resulted in a dramatic increase in p-STAT3 Y705. Combinatorial treatment with pacritinib and TMZ resulted in the abrogation of activated STAT3.

TMZ is currently the standard of care chemotherapy for GBM and is widely used for most GBM patients. Therefore, it is likely that any novel drugs will be tested in combination with TMZ or in TMZ-failed patients. We next asked whether pacritinib would affect the response of BTICs to TMZ. To assess BTIC sensitivity to the combination of pacritinib and TMZ, a clinically relevant dose of TMZ (10 μg/mL) [28] was tested in combination with a suboptimal dose of 1 μM pacritinib. BTIC cultures with different *MGMT* methylation status (S1 Table) were tested. The *MGMT* methylated BTIC cultures were highly sensitive to TMZ. Importantly, pacritinib did not change the effectiveness of TMZ in these methylated cultures (representative BTIC cultures, BT67 and BT69, shown; Fig 2A). Further, in no instance was the combination of pacritinib and TMZ less effective than TMZ alone (Fig 2A and 2B). *MGMT* unmethylated BTIC cultures were largely resistant to TMZ, but responded to the combination of suboptimal doses of pacritinib and TMZ (representative line BT12 shown; Fig 2B). For BT12, a dose of 1 μM pacritinib in combination with 10 μg/mL TMZ, was significantly more effective at reducing BTIC viability than either agent alone (Fig 2B). There was no effect on normal human astrocytes at the concentrations tested (Fig 2C). Further, there was minimal percent inhibition in response to 1 μM pacritinib or 10 μg/mL TMZ alone; however, the combination was synergistic. For BT12, from bliss independence, synergy was observed with a combination effect above 15.97% (Fig 2D). This synergistic effect between pacritinib and TMZ was confirmed on another line, BT53 (Fig 2D). Further, through western blotting analysis, we observed a dramatic increase in activated STAT3 (p-STAT3 Y705) upon TMZ treatment (Fig 2E). It has been previously shown that STAT3 inhibition overcomes TMZ-resistance by downregulating MGMT [29]. Here, we identify the activation of STAT3 in response to TMZ treatment as a potential compensatory mechanism that may play a role in TMZ resistance. Combination treatment with pacritinib and TMZ resulted in an abrogation of this compensatory increase in STAT3 signaling (Fig 2E), providing a possible explanation for the synergy observed between pacritinib and TMZ. This data suggests that pacritinib is compatible with TMZ chemotherapy and may even potentiate its effect.

### Pacritinib inhibits STAT3 activation with minimal effects on other signaling pathways

**Fig 3:**
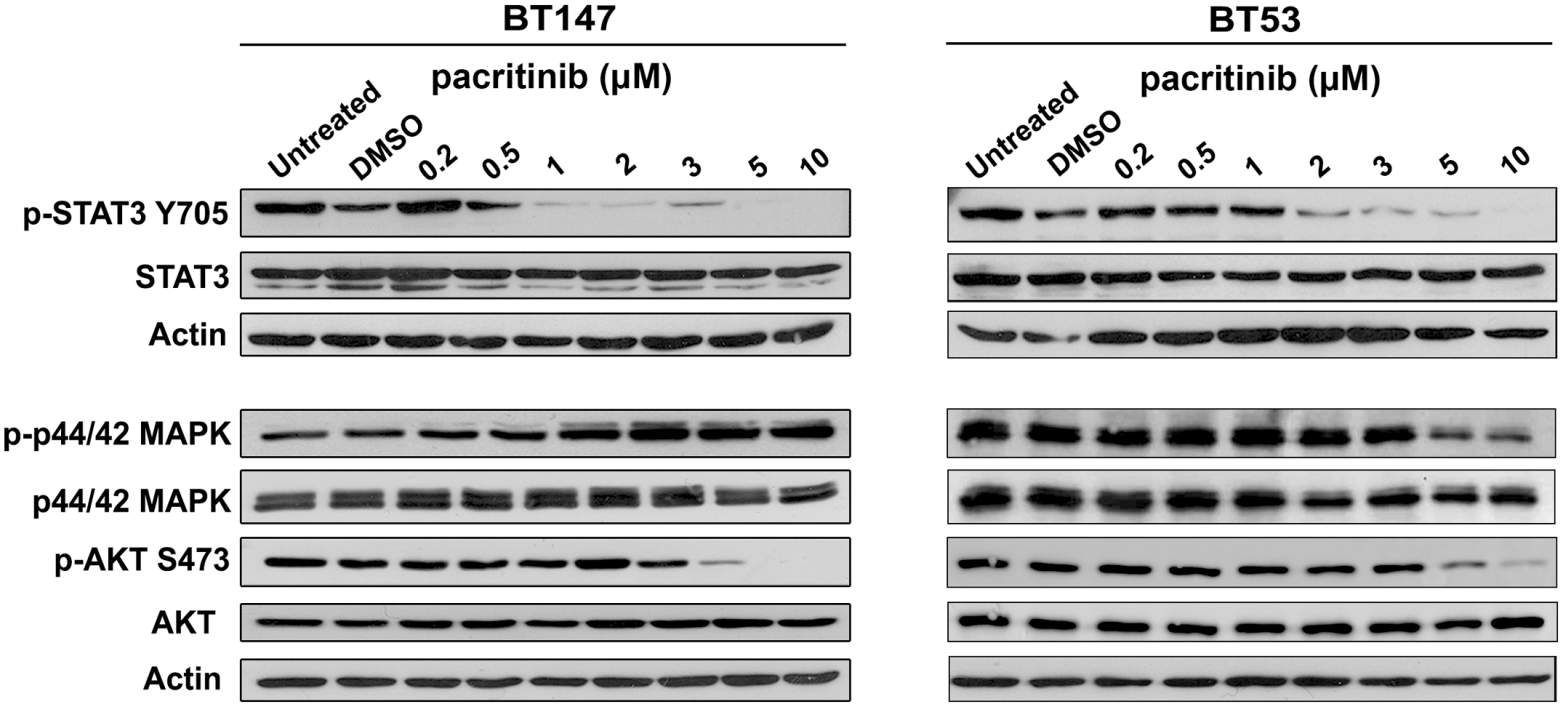
Pacritinib inhibits STAT3 with minimal effects on other signaling pathways. Low micromolar concentrations of pacritinib reduced phosphorylation of tyrosine 705 of STAT3 in BT147 and BT53 at 3 hours, but did not affect phosphorylation of p44/42 MAPK or AKT S473. Pacritinib reduced phosphorylation of AKT S473 and p44/42 MAPK at higher concentrations (5 μM and 10 μM).

We next asked whether pacritinib demonstrated specificity for reducing JAK2 mediated STAT3 activity. We performed western blotting to evaluate its effects on JAK2/STAT3 and other kinase signaling pathways. In BT147, a dose of 1 μM pacritinib strongly decreased STAT3 activation, as demonstrated by the decrease in STAT3 phosphorylation on tyrosine residue 705 (Fig 3). Similarly, in BT53, low micromolar concentrations of pacritinib decreased STAT3 activation (Fig 3). Moreover, at these concentrations, pacritinib did not impact other signaling pathways, as indicated by unchanged levels of phosphorylation on p44/42 MAPK (T202/Y204) and AKT S473. At higher concentrations of pacritinib (5 and 10 μM), p-Akt S473 was decreased in both BT147 and BT53. Further, in BT53, treatment with 5 and 10 μM pacritinib resulted in decreased p-p44/42 MAPK. This suggests potential off-target activities at higher concentrations of pacritinib (Fig 3).

### Pacritinib penetrates the BBB and accumulates in the brains of SCID mice; however, failed to provide a survival advantage possibly due to its rapid metabolism *in vivo*

Despite demonstrating efficacy *in vitro*, often inhibitors fail to demonstrate effectiveness *in vivo*, due to limited BBB penetration. We therefore asked whether pacritinib penetrated the BBB. Pacritinib was delivered, for five days, at 100 and 200 mg/kg via oral gavage, and serum was collected at 30 and 300 minutes. Brains were collected at the 300-minute time point. Concentrations of pacritinib in the serum and brain were determined by LC-MS. The serum concentration of pacritinib increased dose dependently at 30 min to 2.66 μM and 12.6 μM with the 100 and 200 mg/kg doses, respectively (Table 1). At 300 minutes, the concentration decreased to ~0.5 μM for both doses indicating rapid clearance (Table 1). The brain concentrations of pacritinib were 0.91 ± 0.061 μM and 0.84 ± 0.020 μM with the 100 and 200 mg/kg doses, respectively (Table 1). Administering a higher dose of pacritinib did not result in increased brain accumulation at 300 minutes. These data indicate that a threshold for brain penetration of pacritinib may have been reached at 100 mg/kg.

**Table 1:** Five-day pharmacokinetic properties of pacritinib.

	Five-day pharmacokinetics			
	100 mg/kg pacritinib		200 mg/kg pacritinib	
Time	[Serum] (μM)	[Brain] (μM)	[Serum] (μM)	[Brain] (μM)
**30 minutes**	2.66 ± 0.72	N/A	12.6 ± 0.98	N/A
**300 minutes**	0.51 ± 0.14	0.91 ± 0.061	0.43 ± 0.12	0.84 ± 0.020

Serum concentration of pacritinib at 30 and 300 minutes post-dosing with 100 or 200 mg/kg of pacritinib. Brain concentration of pacritinib at 300 minutes post-dosing. Values represent mean ± SD. Sample size is N = 3.

**Fig 4:**
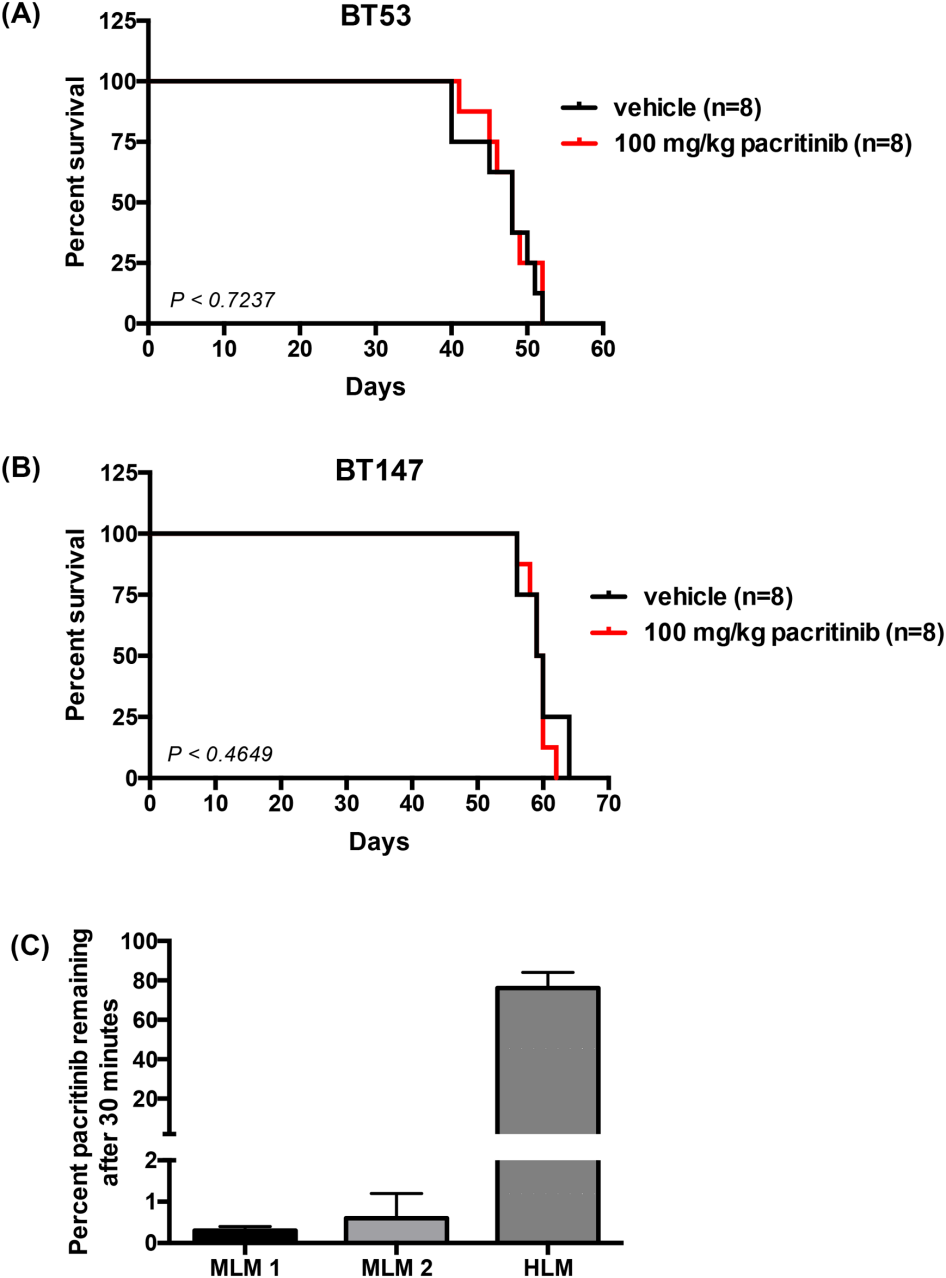
Pacritinib failed to provide a survival advantage possibly due to its rapid metabolism *in vivo*. Kaplan-Meier survival curves showing no survival benefit from treatment with 100 mg/kg pacritinib in mouse orthotopic xenograft models of (A) BT53 (p < 0.7237; log-rank test) and (B) BT147 (p < 0.4649; log-rank test). (C) In mouse liver microsomes (MLM), pacritinib is rapidly metabolized, while in human liver microsomes (HLM), 76% of pacritinib remains after the 30-minute incubation period. Error bars represent SD.

After demonstrating stable pharmacokinetics properties, we asked whether pacritinib could provide a survival advantage to tumor bearing mice. 16 mice were xenografted with 5 x 10^4^ BT53 cells each, and then randomized into pacritinib (n = 8) or vehicle (n = 8) cohorts. Treatment began one week post-xenografts and the mice were treated for three weeks, five times a week. Pacritinib failed to provide a survival advantage following three weeks of treatment (p < 0.7237; log-rank test) (Fig 4A). This was repeated on another BTIC, BT147, and again no survival advantage was obtained (p < 0.4649; log-rank test) (Fig 4B). Despite trials on different BTICs (S2 Fig) and different dosing regimens, pacritinib as a single agent did not provide a survival advantage in treated mice.

We next considered whether pacritinib metabolism differed between mice and humans. Promising pharmacokinetic properties of pacritinib have been previously reported in humans obtained from phase 1/2 studies in myelofibrosis [30], hence we wanted to further study the pharmacokinetic properties of pacritinib in our mouse model. To investigate the clearance of pacritinib, we used mouse and human liver microsomes to compare drug metabolism [31]. We observed a significant interspecies difference in the way pacritinib was metabolized. Following 30-minute incubation with mouse liver microsomes, < 1% pacritinib remained (Fig 4C and S2 Table). However, with human liver microsomes, 76.2 ± 7.9% remained following the 30-minute incubation period (Fig 4C and S2 Table). Pacritinib was rapidly metabolized by mouse liver microsomes, but not human liver microsomes. These results indicate that the lack of efficacy seen in the survival study may be due, in part, to the rapid metabolism of pacritinib in mice.

### Pacritinib in combination with TMZ improves overall median survival

**Fig 5:**
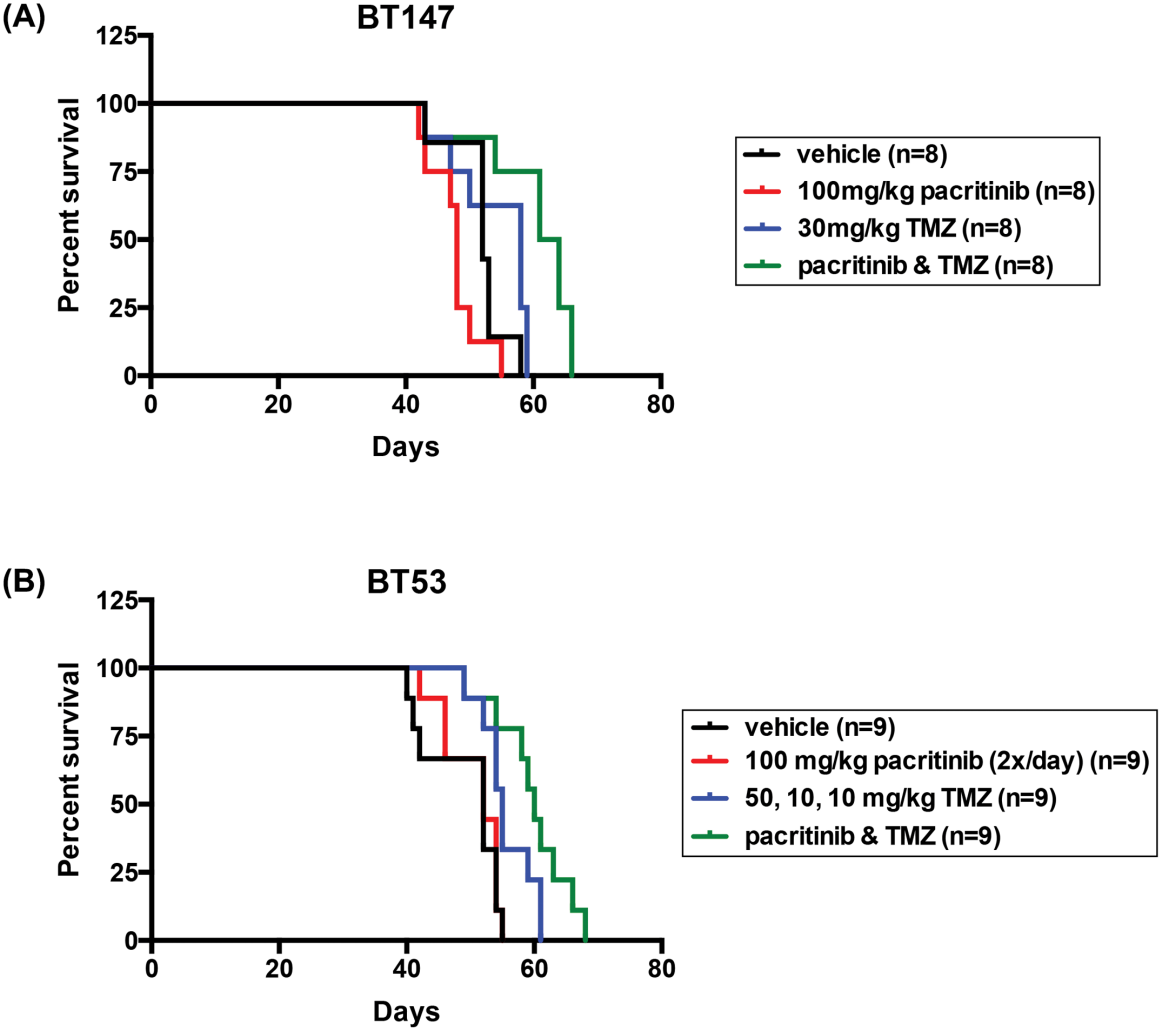
Combination treatment with pacritinib and TMZ improved median overall survival in orthotopic BTIC mouse models. (A) Systemic administration of 100 mg/kg pacritinib in combination with 30 mg/kg TMZ thrice weekly for five consecutive weeks significantly increased median overall survival of BT147 xenografted animals compared to either agent alone. The combination of pacritinib and TMZ resulted in a median survival of 62.5 days compared to a median survival of 52 days in the control cohort, 48 days in the pacritinib cohort, and 58 days in the TMZ cohort. (B) For BT53, 100 mg/kg of pacritinib was administered twice per day alone or in combination with 50 mg/kg TMZ for one week. The following two weeks, mice received 100 mg/kg pacritinib twice per day in combination with 10 mg/kg TMZ. This three-week treatment regimen significantly increased median overall survival of BT53 xenografted animals. The combination of pacritinib and TMZ resulted in a median survival of 60 days compared to a median survival of 52 days in the control cohort.

TMZ is the standard GBM chemotherapy and novel drugs will likely be tested as adjuvants to this regimen. Given the observed synergy between pacritinib and TMZ *in vitro*, we next investigated whether pacritinib in combination with TMZ could improve overall median survival. Thirty-two mice were xenografted with 5 x 10^4^ BT147 cells each and randomized into treatment cohorts. Treatment began one week post cell implantation with mice randomized to vehicle (Ora-Plus), pacritinib (100mg/kg), TMZ (30mg/kg), or pacritinib (100 mg/kg) + TMZ (30 mg/kg) cohorts. Mice were treated for five weeks, three times per week for a total of 15 treatments. The combination of pacritinib and TMZ provided a significant improvement to overall median survival (Fig 5A). Not only was the combination treatment significant over the control (p < 0.0033; log-rank test), it was also significant over the TMZ only cohort (p < 0.0099; log-rank test), and the pacritinib only cohort (p < 0.0001; log-rank test) (Fig 5A). The combination of pacritinib and TMZ resulted in a median survival of 62.5 days compared to a median survival of 52 days in the control cohort, 48 days in the pacritinib cohort, and 58 days in the TMZ cohort (Fig 5A). Smaller tumors were observed in mice that received combination treatment with pacritinib and TMZ than in mice that received vehicle or a single agent (S3 Fig). These data confirm that pacritinib and TMZ are synergistic *in vivo* even in a highly aggressive *MGMT*-unmethylated/*EGFRvIII/PTEN/TP53* mutant line. We also investigated the potential of combination treatment in an *MGMT* methylated line, BT53. Similarly, there was a significant overall median survival advantage in the combination arm over the control (p < 0.0023; log-rank test) (Fig 5B). However, because of the *MGMT* methylation status, BT53 was responsive to TMZ alone, resulting in a significant survival advantage over the control arm (p < 0.0229; log-rank test) (Fig 5B). Importantly, pacritinib in combination with TMZ further increased the median overall survival (Fig 5B). These data support the further investigation of this combination regimen as it demonstrates considerable potential for clinical translation for the treatment of GBM.

## Discussion

GBM is a devastating disease with limited treatment options. We and others, have identified the JAK2/STAT3 signaling pathway as a major targetable oncogenic signaling hub in GBM [9,14,17]. The JAK2/STAT3 pathway plays an important role in proliferation, self-renewal, and maintenance of multipotency in GBM BTICs [32] and pluripotency in embryonic stem cells [33,34]. It also has an important role in regulating differentiation of normal neural stem cells and thus has an essential role in the development of the nervous system [35]. JAK2/STAT3 signaling becomes deregulated in most cancers and is over activated in BTICs and GBM [9,10]. Despite our improved understanding of the important role JAK2/STAT3 signaling plays in GBM pathogenesis, JAK2/STAT3 inhibitors have yet to be successfully translated to the clinic.

Pacritinib, a novel JAK2 inhibitor with promising pharmacological properties [21,30,36], shows promise for the treatment of GBM. Here, we show that JAK2/STAT3 inhibition with pacritinib is highly effective against BTICs *in vitro*, and not influenced by the status of common GBM molecular alterations including *MGMT* promoter methylation, *EGFR*, *PTEN*, *TP53*, *NF1*, and *CDKN2A* mutations. This is consistent with our previous study using other JAK2 inhibitors [17]. Further, pacritinib demonstrated synergy with TMZ, the current standard of care therapy for GBM. TMZ is only effective in a subset of patients with *MGMT* methylated GBMs [1,4]. Our results suggest that combined treatment with pacritinib and TMZ may be effective against a larger population of patients with diverse molecular heterogeneity, similar to what is observed in the BTICs. The combination of pacritinib and TMZ proved to be synergistic in BTICs that did not respond to TMZ alone. This highlights the possibility that pacritinib could augment TMZ sensitivity in patients that do not normally respond to the standard of care. Importantly, pacritinib did not attenuate the sensitivity of TMZ-responsive BTIC cultures. Further, we observed a dramatic increase in activated STAT3 upon treatment with TMZ. It has been previously shown that STAT3 inhibition overcomes TMZ resistance by downregulating MGMT [29]. This study demonstrated that the activation of JAK2/STAT3 signaling following treatment with TMZ is a potential mechanism of TMZ resistance in GBM BTICs. Combined treatment with pacritinib and TMZ dramatically reduced the activity of the JAK2/STAT3 pathway. This highlights the potential for pacritinib to be a useful adjuvant therapy with the standard of care TMZ. Additionally, pacritinib could be used as a salvage therapy for patients with a TMZ resistant recurrent disease, as STAT3 inhibition sensitizes TMZ resistant BTIC cultures.

A major challenge with investigating new treatment strategies for GBM is finding drugs that effectively penetrate the BBB. Here, we show that not only is pacritinib effective *in vitro*, it also demonstrates potential *in vivo* as it effectively penetrates the BBB. However, we observed a significant interspecies difference in the metabolism of pacritinib using mouse and human liver microsomes. It has been previous reported that the relative amount of hepatic drug metabolizing enzymes are higher in mice than in humans [37,38]. The liver is the most important organ for drug metabolism [31] and pacritinib was rapidly metabolized by mouse but not by human liver microsomes. The latter is consistent with pharmacokinetic studies of pacritinib in myelofibrosis patients [30]. In a phase 1/2 study of pacritinib in patients with myelofibrosis, one day treatment with 100 mg of pacritinib resulted in a mean maximum concentration (C_max_) of 7.8 μM (3699 ng/mL). The median time to maximum concentration (T_max_) was 5 hours [30]. Conversely, in our mouse model, following five days of treatment, the serum concentration of pacritinib at 30 minutes was only 2.66 μM for treatment with 100 mg/kg. Five hours post-treatment, the concentration of pacritinib detected in the serum decreased to 0.51 μM, indicating rapid clearance. This suggests that pacritinib may be more effective in humans as it displays more favorable pharmacokinetic properties in humans compared to mice. Notwithstanding its rapid metabolism, pacritinib was still detectable in the brains of non-tumor bearing mice, indicating that pacritinib can cross the BBB. It is also possible that pacritinib may have improved results in humans, where the drug is not rapidly metabolized and may be able to penetrate the brain at much higher concentrations allowing for a sustained therapeutic effect. Pacritinib failed to improve the median overall survival as a monotherapy in mice, likely due to its rapid metabolism; however, when it was co-administered with TMZ, there was a significant increase in overall median survival. This was especially impressive given the highly aggressive nature of BT147, the *MGMT*-unmethylated/*EGFRvIII*/*PTEN*/*TP53* mutant line used for xenograft experiments. This data suggests that the low micromolar accumulation of pacritinib in the brain was sufficient to synergize with TMZ and improve overall survival.

It was evident that co-administration of pacritinib and TMZ was beneficial throughout the course of treatment; nevertheless, termination of treatment resulted in the rapid deterioration of the mice. Our results suggest that the combinatorial treatment can prevent the rapid growth of the tumor, but cannot efficiently kill all of the BTICs. This begs the question as to whether a continuous, long-term treatment regimen would be more successful. Continuous treatment in rodents was not tolerated for more than five weeks, as treatment of mice xenografted with BT147 had to be stopped due to toxicities including weight loss and lack of grooming. We hypothesize that there would have been further survival benefit had continuous dosing been possible. It remains to be investigated whether GBM patients could tolerate long-term JAK2/STAT3 inhibition, without significant side effects. Concerns with JAK inhibitors include myelosuppressive effects as well as immunosuppression. However, previous clinical studies with pacritinib for other indications support the tolerability of the drug in humans. Pacritinib has already completed phase 3 clinical trials for myelofibrosis [21]. Data obtained from these early trials support the long term tolerability of pacritinib (reviewed in [39]). The most frequent adverse side effects were gastrointestinal; however, these side effects are manageable and rarely resulted in the termination of the treatment [36]. The tolerability of pacritinib is thought to be due to its specificity to JAK2 and its lack of JAK1 inhibition [40]. JAK1 is important for a healthy immune response, including IL2 and interferon signaling, which is blocked when JAK1 is inhibited [41]. Pacritinib has not been used in GBM and therefore, its use would require careful monitoring, as with most other chemotherapy regimens, to minimize and treat any potential side effects. The risks associated with drugs that may alter immune function are well understood. With these types of inhibitors, there are risks of reactivation of latent infections, decreased ability to fight off new infections, and increased risk of certain neoplasms [42]. However, given the universally dismal prognosis for GBM patients, achieving improved tumor control and improving quality of life would almost certainly outweigh these risks.

Pacritinib is one of the few JAK2/STAT3 inhibitors with BBB-permeability and efficacy against intracranial tumors at low micromolar doses. Importantly, when combined with TMZ, the current standard of care for GBM, it significantly improved the median overall survival in an orthotopic BTIC xenograft mouse model, making it a promising compound for GBM treatment. Given the promising safety profiles of pacritinib in humans and the fact that it has already completed a Phase 3 trial for myelofibrosis, progression to an early phase trial for GBM would be a feasible endeavour, should further preclinical evaluation continue to yield positive results. As such, compounds such as pacritinib may have the potential to further extend the survival of GBM patients when combined with the current standard of care.

## Supporting information

S1 Fig Quantification of decreased sphere formation in a dose dependent manner in a diverse panel of BTICs. Number of spheres per well normalized to DMSO shown for 8 BTIC cultures. Pacritinib completely abolished sphere formation by 5 μM in all BTICs tested (**** denotes p < 0.0001 vs untreated; ANOVA).
(TIF)

S2 Fig Pacritinib failed to provide a survival advantage as a monotherapy. (A) Kaplan-Meier survival curves showing no efficacy of pacritinib treatment alone in mouse orthotopic xenograft models of BT143 (p < 0.4622; log-rank test) and BT206 (p < 0.0968; log-rank test). (B) Body weights were monitored over the course of treatment.
(TIF)

S3 Fig Combinatorial inhibition reduced tumor burden in orthotopically xenografted BT53 mice. Immunostaining for human nucleolin revealed that treatment with 30 mg/kg TMZ and 100 mg/kg pacritinib resulted in smaller tumors compared to the mice that received either agent alone.
(TIF)

S1 Table Identification of a diverse panel of GBM BTICs representative of the different mutational statuses of GBM patients. (DOCX)

S2 Table Human and mouse liver microsome analysis with pacritinib and test compounds. (DOCX)

S1 File ARRIVE Guidelines Checklist (Jensen et al. 2017). (PDF)
