# Supplementary figures and images for: The JAK2/STAT3 inhibitor pacritinib effectively inhibits patient-derived GBM brain tumor initiating cells in vitro and when used in combination with temozolomide increases survival in an orthotopic xenograft model
Source: PLoS One. 2017 Dec 18;12(12):e0189670. doi: 10.1371/journal.pone.0189670 (PMC5734728; doi:10.1371/journal.pone.0189670)

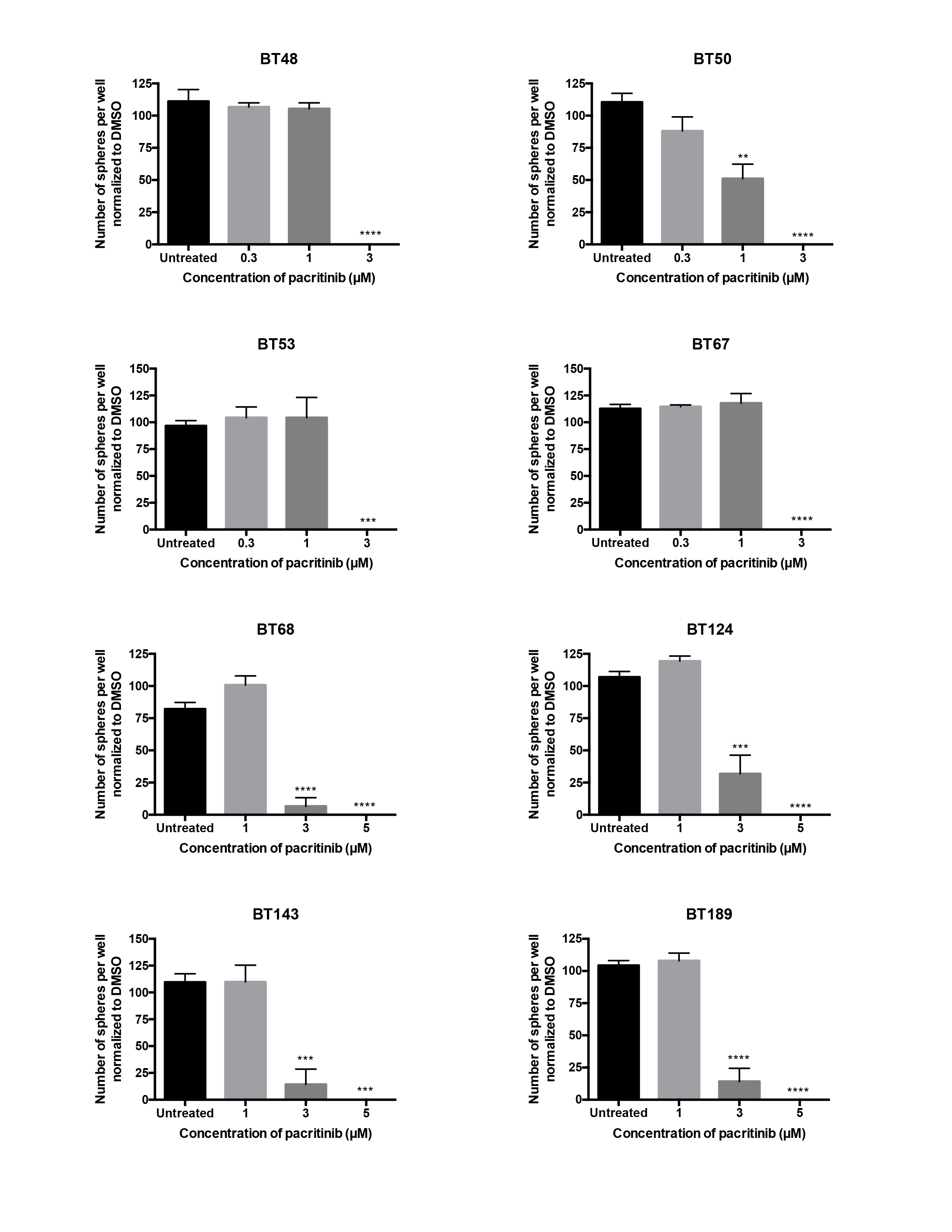

Supplement: S1 Fig — Number of spheres per well normalized to DMSO shown for 8 BTIC cultures. Pacritinib completely abolished sphere formation by 5 μM in all BTICs tested (**** denotes p < 0.0001 vs untreated; ANOVA). (TIF) [file pone.0189670.s001.tif]

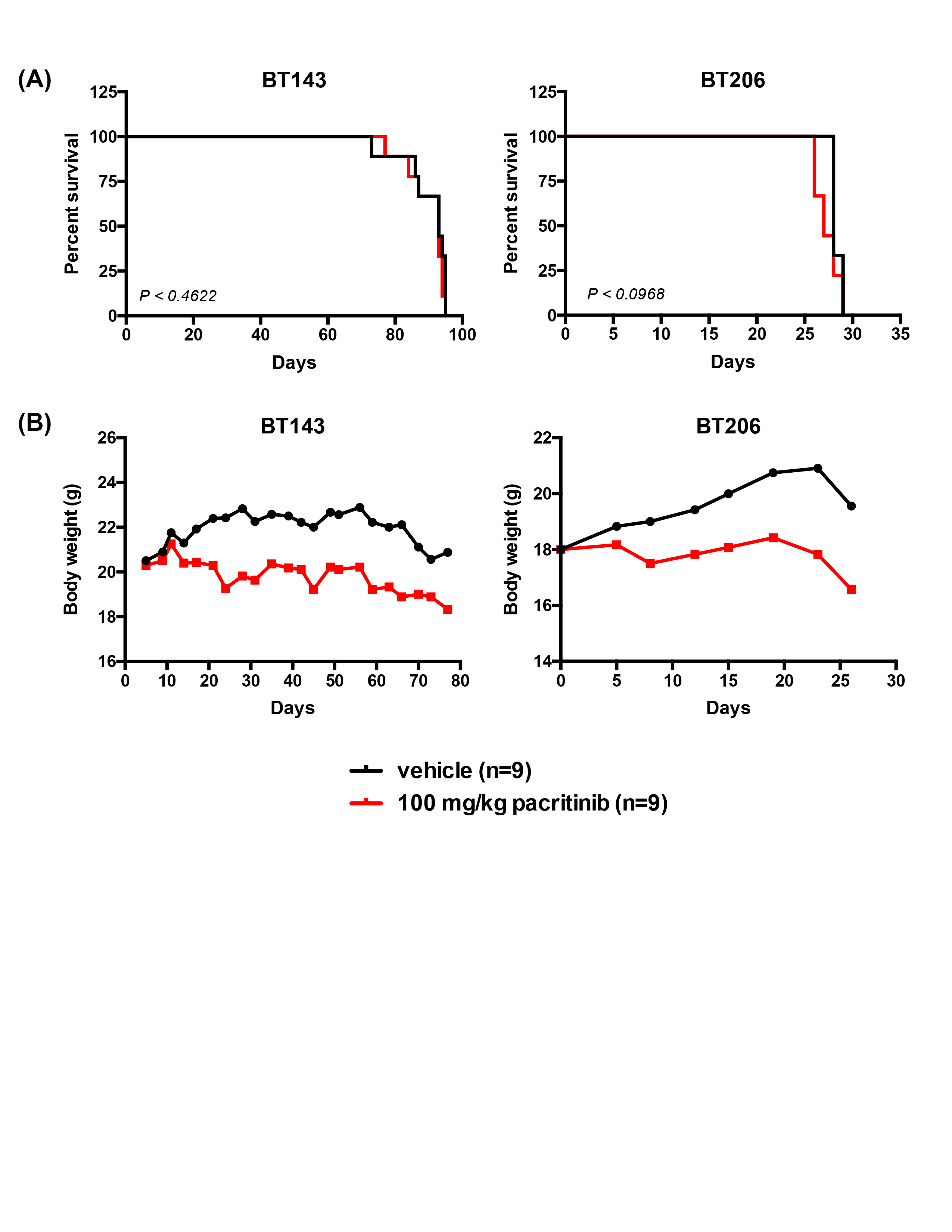

Supplement: S2 Fig — (A) Kaplan-Meier survival curves showing no efficacy of pacritinib treatment alone in mouse orthotopic xenograft models of BT143 (p < 0.4622; log-rank test) and BT206 (p < 0.0968; log-rank test). (B) Body weights were monitored over the course of treatment. (TIF) [file pone.0189670.s002.tif]

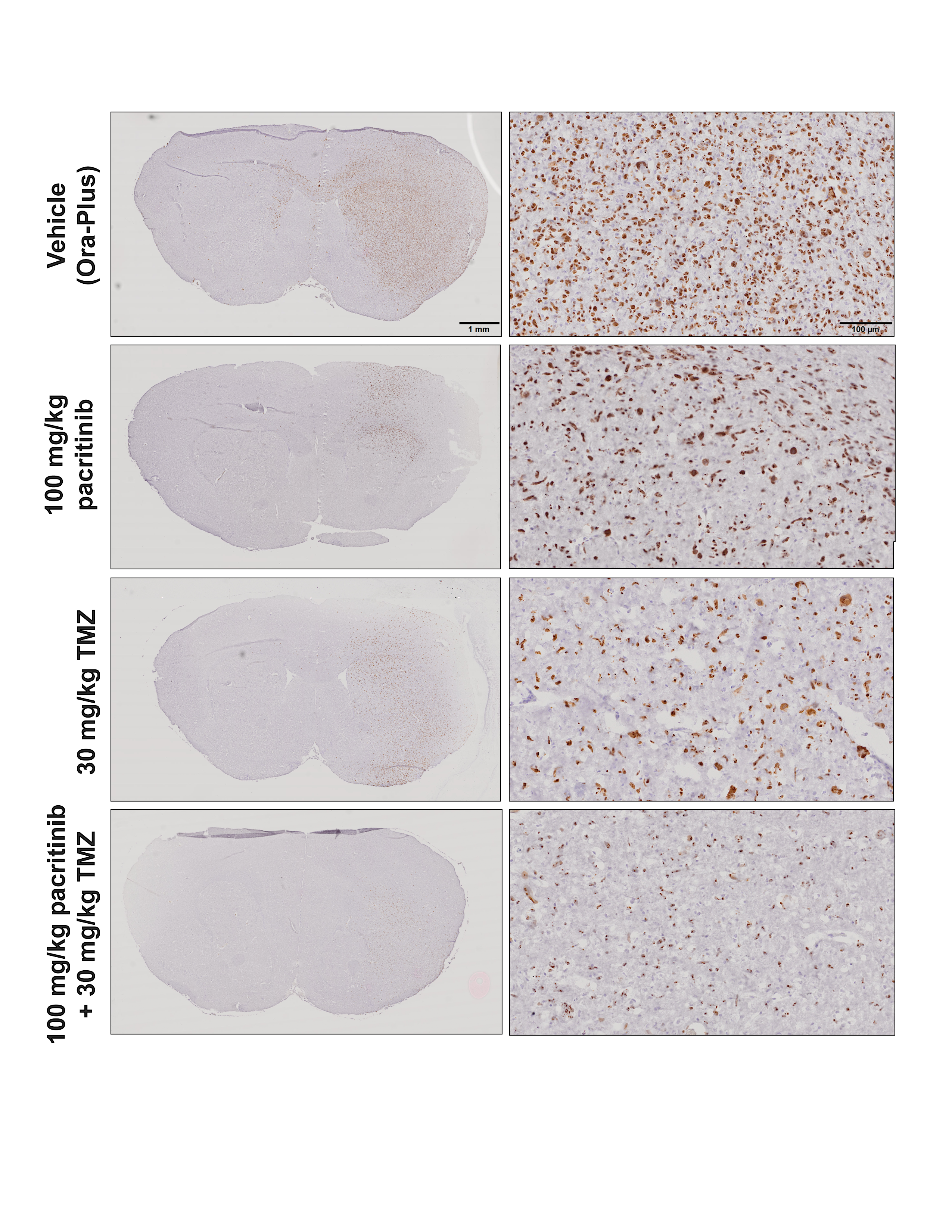

Supplement: S3 Fig — Immunostaining for human nucleolin revealed that treatment with 30 mg/kg TMZ and 100 mg/kg pacritinib resulted in smaller tumors compared to the mice that received either agent alone. (TIF) [file pone.0189670.s003.tif]
