# Supplementary material for: The JAK2/STAT3 inhibitor pacritinib effectively inhibits patient-derived GBM brain tumor initiating cells in vitro and when used in combination with temozolomide increases survival in an orthotopic xenograft model
Source: PLoS One. 2017 Dec 18;12(12):e0189670. doi: 10.1371/journal.pone.0189670 (PMC5734728; doi:10.1371/journal.pone.0189670)
Supplement: S1 Table — (DOCX) [file pone.0189670.s004.docx]

**S1 Table. Identification of a diverse panel of GBM BTICs representative of the different mutational statuses of GBM patients.**

Common GBM molecular alterations including *MGMT* promoter methylation*, EGFR*, *PTEN*, *TP53*, *NF1*, *IDH1*, and *CDKN2A* mutation statuses for the eleven BTIC cultures used in this study. Mut indicates mutant and wt indicates wild-type. U indicates unmethylated and M indicated methylated. U/M indicates hemi-methylation. vIII indicates *EGFR* variant III, an activating deletion characteristic of GBM, het del indicates a heterozygous deletion, homo del indicates a homozygous deletion, N/A indicates not available.
