# Supplementary material for: The JAK2/STAT3 inhibitor pacritinib effectively inhibits patient-derived GBM brain tumor initiating cells in vitro and when used in combination with temozolomide increases survival in an orthotopic xenograft model
Source: PLoS One. 2017 Dec 18;12(12):e0189670. doi: 10.1371/journal.pone.0189670 (PMC5734728; doi:10.1371/journal.pone.0189670)
Supplement: S2 Table — (DOCX) [file pone.0189670.s005.docx]

**S2 Table. Human and mouse liver microsome analysis with pacritinib and test compounds.**

| **Mouse Liver Microsomes** | | | |
| --- | --- | --- | --- |
| **Compounds** | **% Remaining** | **STDEV** |  |
| **Pacritinib (run 1)** | 0.3 | 0.1 | Unstable |
| **Pacritinib (run 2)** | 0.6 | 0.6 | Unstable |
| **QC1** | 53.8 | 1.7 | Usual value: 55 ± 10% |
| **Antipyrine** | 96.5 | 5.7 | Usual value: >90% |
| **Propranolol** | 9.6 | 0.8 | Usual value: <20% |

| **Human Liver Microsomes** | | | |
| --- | --- | --- | --- |
| **Compounds** | **% Remaining** | **STDEV** |  |
| **Pacritinib** | 76.2 | 7.9 | Stable |
| **QC1** | 23.3 | 0.7 | Usual value: 25 ± 10% |
| **DXM** | 75.0 | 1.7 | Usual value: 72 ± 10% |

Percentage of the compound remaining following a 30-minute incubation with mouse of human liver microsomes. Pacritinib and control compounds, with their usual values, are indicated in the table.
